# Utilizing AI for the Identification and Validation of Novel Therapeutic Targets and Repurposed Drugs for Endometriosis

**DOI:** 10.1002/advs.202406565

**Published:** 2024-12-12

**Authors:** Bonnie Hei Man Liu, Yuezhen Lin, Xi Long, Sze Wan Hung, Anna Gaponova, Feng Ren, Alex Zhavoronkov, Frank W. Pun, Chi Chiu Wang

**Affiliations:** ^1^ Insilico Medicine Hong Kong Ltd. Unit 310, 3/F, Building 8W, Hong Kong Science and Technology Park Hong Kong China; ^2^ Department of Obstetrics and Gynaecology The Chinese University of Hong Kong Hong Kong China; ^3^ Insilico Medicine Shanghai Ltd. 9F, Chamtime Plaza Block C, Lane 2889, Jinke Road, Pudong New Area Shanghai 201203 China; ^4^ Buck Institute for Research on Aging 8001 Redwood Blvd. Novato CA 94945 USA; ^5^ Reproduction and Development Li Ka Shing Institute of Health Sciences The Chinese University of Hong Kong Hong Kong China; ^6^ School of Biomedical Sciences The Chinese University of Hong Kong Hong Kong China; ^7^ Chinese University of Hong Kong‐Sichuan University Joint Laboratory in Reproductive Medicine The Chinese University of Hong Kong Hong Kong China; ^8^ State Key Laboratory of Chinese Medicine Modernization Innovation Center of Yangtze River Delta Zhejiang University Jiaxing 314102 China

**Keywords:** artificial intelligence, drug repurposing, endometriosis, GBP2, HCK, ITGB2, lifitegrast, target discovery

## Abstract

Endometriosis affects over 190 million women globally, and effective therapies are urgently needed to address the burden of endometriosis on women's health. Using an artificial intelligence (AI)‐driven target discovery platform, two unreported therapeutic targets, guanylate‐binding protein 2 (GBP2) and hematopoietic cell kinase (HCK) are identified, along with a drug repurposing target, integrin beta 2 (ITGB2) for the treatment of endometriosis. GBP2, HCK, and ITGB2 are upregulated in human endometriotic specimens. siRNA‐mediated knockdown of GBP2 and HCK significantly reduced cell viability and proliferation while stimulating apoptosis in endometrial stromal cells. In subcutaneous and intraperitoneal endometriosis mouse models, siRNAs targeting GBP2 and HCK notably reduced lesion volume and weight, with decreased proliferation and increased apoptosis within lesions. Both subcutaneous and intraperitoneal administration of Lifitegrast, an approved ITGB2 antagonist, effectively suppresses lesion growth. Collectively, these data present Lifitegrast as a previously unappreciated intervention for endometriosis treatment and identify GBP2 and HCK as novel druggable targets in endometriosis treatment. This study underscores AI's potential to accelerate the discovery of novel drug targets and facilitate the repurposing of treatment modalities for endometriosis.

## Graphical abstract

Endometriosis is a chronic inflammatory gynecological disease affecting over 190 million women globally, and no curative treatment is currently available. This study identifies two novel therapeutic targets (GBP2 and HCK) through an AI‐driven target discovery platform. This analysis also reveals lifitegrast, an existing drug, as a potential treatment. Experimental validations confirm these AI‐derived findings as new opportunities for endometriosis treatment.

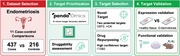

## 1 Introduction

The drug development pipeline has been fundamentally transformed by artificial intelligence (AI) integration, which now influences several aspects, including target identification, novel compound generation, and clinical trial outcome analyses, among others.^[^ ^1^, ^2^ ^]^ Traditional target discovery methods are often hampered by complexity and high costs.^[^ ^3^, ^4^ ^]^ AI addresses these challenges by accelerating target selection through sophisticated algorithms like natural language processing, deep learning models, and generative adversarial networks. These technologies enhance workflows related to disease biology evaluation, omics analysis, and druggability assessments.^[^ ^5^, ^6^, ^7^, ^8^ ^]^ By uncovering patterns in vast biomedical datasets, AI has facilitated significant advancements in target identification, indication prioritization, and clinical trial optimization.^[^ ^9^ ^]^ For instance, validating AI‐assisted identification of novel targets for amyotrophic lateral sclerosis (ALS) using a Drosophila model has provided new insights into ALS pathophysiology and highlighted potential opportunities for therapeutic interventions.^[^ ^10^ ^]^ Additionally, recent studies have demonstrated that AI can significantly reduce the time and cost of identifying promising therapeutic targets in aging and age‐related diseases.^[^ ^11^ ^]^ Notably, a comprehensive AI‐driven analysis has identified several novel dual‐purpose target candidates for treating both cancer and aging, underscoring AI's potential to revolutionize therapeutic strategies across multiple domains.^[^ ^12^ ^]^ AI has also shown promise in drug repurposing, where it aids in identifying new therapeutic uses for existing drugs by analyzing extensive biomedical literature and clinical trial data.^[^ ^8^ ^]^ The emergence of AI‐derived drug candidates in clinical studies further underscores AI's transformative potential in revolutionizing drug development.^[^ ^13^ ^]^ Therefore, the current study employs AI to identify novel therapeutic targets and repurpose drugs to treat endometriosis, aiming to enhance therapeutic precision and efficacy.

Endometriosis is a chronic, inflammatory gynecological disease characterized by the presence of ectopic endometrial glands and stroma, most commonly found on the pelvic peritoneum, ovaries, rectovaginal septum, and in other abdominal and extra‐pelvic locations throughout the body. About 5–15% of women at reproductive age are estimated to suffer from endometriosis^[^ ^14^ ^]^ and its prevalence can reach 35–80% in women with pelvic pain and infertility.^[^ ^15^, ^16^ ^]^ Owing to the variability in symptoms and difficulty in distinguishing endometriosis signs from other disorders, delayed diagnosis, with the means ranging from 4–12 years, is a common problem.^[^ ^17^, ^18^, ^19^, ^20^ ^]^ The etiology of endometriosis remains obscure, and several hypotheses have been proposed to explain its origin.^[^ ^8^ ^]^ Genetics, environment, immune dysfunction, and estrogen perturbation may contribute to the pathology of endometriotic lesions.^[^ ^21^ ^]^ Among these, dysregulated steroidogenesis is one commonly studied mechanism, emphasizing hormonal therapy as an effective treatment for the disease.

Due to the heterogeneous nature of its pathogenesis, endometriosis remains incurable. Multiple therapeutic approaches, such as surgery, pharmacological treatment, and acupuncture, have been adopted to manage endometriosis and its comorbidities. Hormone therapy is regarded as first‐line therapy for endometriosis. Progestins (medroxyprogesterone acetate, norethisterone acetate, cyproterone acetate, dienogest) and gonadotrophin‐releasing hormone analogues (nafarelin, leuprorelin, buserelin, goserelin or triptorelin), two approved hormonal medications for endometriosis, demonstrated promising efficacy in reducing endometriosis‐associated pain without significant safety concerns,^[^ ^22^, ^23^, ^24^, ^25^, ^26^ ^]^ but their long‐term effects still remain unknown. The chronic pain and infertility caused by endometriosis adversely impact women's quality of life, and its economic burden is estimated to reach $80 billion annually in the US alone.^[^ ^27^ ^]^ Moreover, mounting evidence suggests endometriosis is a systemic disease that disturbs cardiovascular, neurological, metabolic, and immune functions.^[^ ^16^ ^]^ Patients may exhibit increased risks of developing several chronic diseases, such as adenomyosis,^[^ ^28^ ^]^ ovarian cancer,^[^ ^29^ ^]^ and autoimmune diseases.^[^ ^30^ ^]^ Thus, a pressing, unmet clinical need exists to develop new and effective therapeutics for endometriosis.

To address this issue, we utilized an AI‐driven target discovery platform, PandaOmics, to identify novel druggable targets and analyze approved drugs that could be repurposed to treat endometriosis. PandaOmics systematically analyzed large‐scale transcriptomic data to rank potential targets based on their dysregulated expression in endometriosis, their predicted druggability, and existing evidence from the literature supporting their roles in driving the pathogenesis of the disease. Identifying GBP2 and HCK as targets without prior text‐based evidence in endometriosis studies highlights the power of our AI‐driven approach in uncovering previously unexplored mechanisms. GBP2 is a guanylate‐binding protein implicated in regulating immune and inflammatory processes,^[^ ^31^, ^32^ ^]^ while HCK is a Src family kinase involved in cell proliferation and survival signaling.^[^ ^33^, ^34^ ^]^ To validate these targets, we conducted targeted knockdown experiments using small interference RNA (siRNA) and demonstrated that inhibition of either GBP2 or HCK suppressed proliferation and stimulated apoptosis of ectopic endometrial cells both in vitro and in vivo, suggesting their potential regulatory roles in immune response and growth of ectopic endometrial tissue in endometriosis. Furthermore, Lifitegrast, an approved ITGB2 inhibitor, was identified as a candidate drug through PandaOmics that may have therapeutic value in treating endometriosis. Indeed, Lifitegrast suppressed endometriotic growth in preclinical murine endometriosis models, demonstrating a novel role for ITGB2 in endometriosis pathology. Together, these data demonstrate the power of AI in accelerating target discovery and repurposing existing therapies for unappreciated indications. The validation of GBP2 and HCK as key regulators and the efficacy of Lifitegrast provide a strong rationale for further investigations.

## 2 Results

### 2.1 Identification of GBP2 and HCK as Potential Novel Therapeutic Targets for Endometriosis

**Figure 1:**
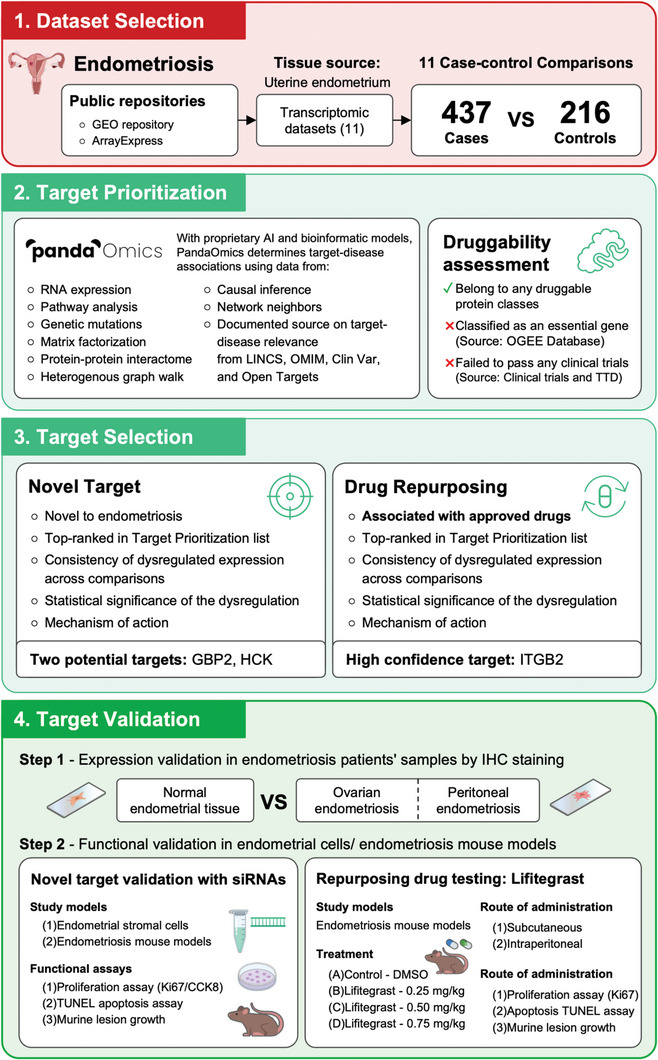
Workflow for identifying therapeutic targets and repurposing drugs for endometriosis using artificial intelligence techniques. 1) Dataset selection: With the input of 11 ectopic endometrium transcriptomic datasets, 11 case‐control comparisons were generated using 437 cases collected from patients with endometriosis and 216 healthy control samples for therapeutic target identification. 2) Target prioritization: With its proprietary AI and bioinformatic models, PandaOmics is a generative AI system that determines target‐disease association and prioritizes targets using the listed sources and characteristics. This prioritization step also integrates a druggability assessment of candidate targets. 3) Target selection: Following the prioritization of targets based on the listed characteristics, GBP2 and HCK were selected as novel targets. In parallel, ITGB2 was nominated as a high‐confidence target for drug repurposing. 4) Target validation: Proposed targets were validated by histology in patient samples and functional validation in preclinical endometriosis models. GEO: Gene Expression Omnibus; LINCS: Library of Integrated Network‐Based Cellular Signatures; OMIM: Online Mendelian Inheritance in Man; OGEE: Online Gene Essentiality; TTD: Therapeutic Target Database.

**Figure** **1** illustrates our study's workflow. With the aid of PandaOmics, we performed a meta‐analysis of endometriosis‐associated datasets based on ectopic endometrium tissues to identify targets. Together with the expression and pathway analyses, we revealed two categories of druggable targets: novel targets and targets with approved drugs for repurposing.

To evaluate the variation of gene expression among different subtypes and uterine cycles of endometriosis, ten case‐control comparisons were generated from the GEO dataset GSE141549. We performed one subtype‐ and cycle‐nonspecific comparison, three subtype‐specific comparisons, and six cycle‐specific comparisons and analyzed expression correlation using differentially expressed gene (DEG) profiles (Table S1, Supporting Information). A high correlation was observed between the ten comparisons (Figure S1A, Supporting Information), indicating the discriminant nature of disease subtype‐ and uterine cycle‐specific samples. To select datasets for target identification, we determined the expression correlation of 12 endometriosis transcriptomic datasets (Table S1, Supporting Information) and excluded GSE7846, which exhibited a negative correlation with 7 (63.6%) out of 11 datasets (Figure S1B, Supporting Information).

**Figure 2:**
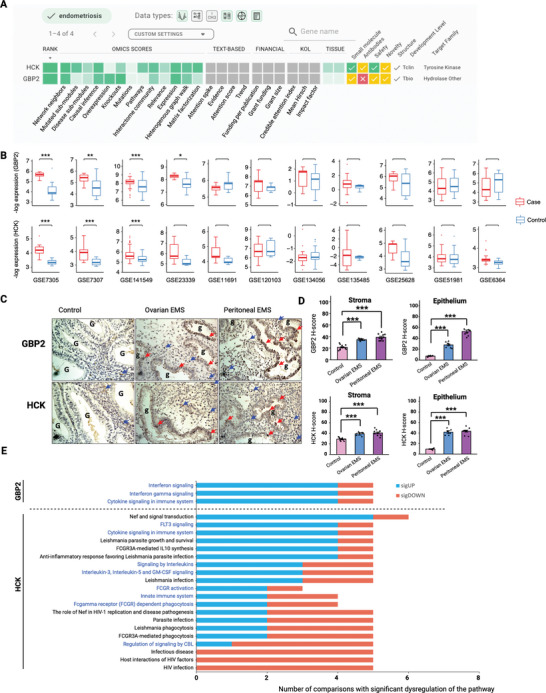
GBP2 and HCK were identified as potential therapeutic targets for endometriosis. A) Screenshot of the Target ID page of PandaOmics for endometriosis meta‐analysis. GBP2 and HCK were revealed as novel druggable targets for endometriosis. B) *GBP2* and *HCK* transcript levels in the eleven ectopic endometrium‐related comparisons were displayed in box plots. *FDR < 0.05, **FDR < 0.01, ***FDR < 0.001. FDR < 0.05 indicates a significant differential expression. C) Representative images of the paraffin‐embedded human endometriosis and normal endometrium tissues stained with anti‐GBP2 and anti‐HCK antibodies. G, endometrium glands; g: endometriotic glands; blue arrows, positive staining in endometrial or endometriotic stromal cells; red arrows, positive staining in endometrial or endometriotic epithelial cells. Magnifications, 40x. D) GBP2 and HCK protein expression in the epithelial and stromal cells was assessed by H‐score. Control (*n* = 10), ovarian EMS (*n* = 7), and peritoneal EMS (*n* = 10). Data are represented as mean ± SEM. *P < 0.05, **P < 0.01, ***P < 0.001. *P* < 0.05 is considered as statistically significant. E) Dysregulated pathways associated with GBP2 (upper) and HCK (lower) in endometriosis comparisons were displayed. Pathways were annotated by the Reactome database, and the iPANDA algorithm determined the degree of pathway dysregulation. Blue and red bar colors indicate the number of comparisons with significant activation or inactivation of the corresponding pathway, respectively. Pathways labeled in blue are immune‐associated.

Based on the expression correlation results, a meta‐analysis with 11 subtype‐ and cycle‐nonspecific comparisons (Table S1, Supporting Information) was conducted in PandaOmics, which provides thirteen Omics and ten text‐based AI models for therapeutic target prioritization.^[^ ^35^ ^]^ 98 unique genes were selected based on PandaOmic's ranking (Tables S2 and S3, Supporting Information), which prioritized the following features: consistency of dysregulated expression across comparisons, statistical significance of the dysregulation, druggability, and literature support for its potential role in endometriosis‐related mechanisms. Based on the aggregated scores from the 13 Omics models, GBP2 (Guanylate Binding Protein 2) and HCK (Hemopoietic Cell Kinase) attained the highest rankings as targets for endometriosis, without prior text‐based evidence from endometriosis studies (Figure S2, Supporting Information). Among the Omics scores, GBP2 and HCK showed predominant performance (scored over 0.7) in the “Network Neighbors”, “Causal Inference”, and “Expression” models (**Figure** **2**A), signifying their potential as novel therapeutic targets for the treatment of endometriosis. Both targets were considered safe as they were not reported as essential genes in the Database of Essential Genes (Figure 2A, Safety filter). HCK was deemed more druggable than GBP2 due to its membrane‐bound kinase nature and the availability of HCK‐associated drugs (Figure 2A, Filters for Small Molecule and Antibodies).

### 2.2 GBP2 and HCK Expression is Upregulated in Human Endometriotic Samples

To validate the AI‐derived target selections, we measured HCK and GBP2 expression in samples collected from the endometriotic lesions and control endometrium using transcriptomic datasets (Table S1, Supporting Information). In 8 out of 11 case‐control comparisons (72.7%), both GBP2 and HCK were upregulated (logFC > 0) in endometriotic lesions relative to control samples. Of these, four comparisons of GBP2 upregulations and three comparisons of HCK upregulation reached statistical significance (FDR < 0.05), as detailed in Figure 2B and Table S4 (Supporting Information). Immunohistochemistry (IHC) staining in the in‐house paraffin‐embedded ovarian and peritoneal endometriotic tissue (EMS) samples corroborated the protein expression dysregulation of the two targets (Figure 2C,D). EMS refers to the abnormal growth of endometrial‐like tissue, consisting of glands and stroma, found outside the uterine cavity in ectopic locations such as the ovaries, fallopian tubes, and peritoneum. This tissue, characteristic of endometriosis, responds to hormonal changes and can cause symptoms like pelvic pain and infertility, often exhibiting altered cellular and molecular characteristics compared to normal endometrium. Compared to stromal and epithelial cells in normal endometrium tissue, GBP2 expression was significantly elevated in both stromal and epithelial cells in ovarian and peritoneal EMS (Figure 2C,D, upper panel). Similarly, HCK expression in stromal and epithelial cells within ovarian and peritoneal EMS tissues was notably higher than in their normal counterparts (Figure 2C,D, lower panel). Consistent upregulations of GBP2 and HCK were observed at mRNA and protein levels in endometriotic samples, supporting their potential roles in driving endometriosis.

Dysregulated pathway analysis was performed to predict the potential functions of GBP2 and HCK in the endometriosis pathogenesis (Figure 2E). The number of transcriptomic comparisons having significant dysregulation of each pathway (*P* < 0.05) was indicated by the length of the bar (blue and red represent activation and inhibition, respectively). All dysregulated pathways involving GBP2 were immune‐associated and significantly upregulated (Figure 2E *upper*; Table S5, Supporting Information). Most pathways associated with HCK were linked to immune function and infection (Figure 2E *lower*; Table S5, Supporting Information), with 4 out of 8 HCK‐associated immune pathways upregulated. These results indicate that GBP2 and HCK are implicated in the immune response and suggest that targeting them may alleviate chronic inflammation associated with endometriosis.

### 2.3 Knockdown of *GBP2* and *HCK* Diminished Endometriosis In Vitro and In Vivo

**Figure 3:**
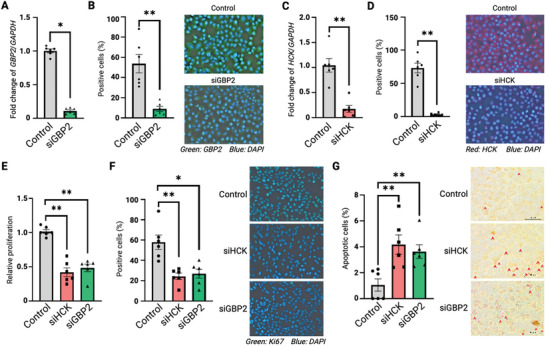
GBP2 and HCK ablation inhibited proliferation and promoted apoptosis of endometriotic stromal cells. Endometriotic stromal cells were transiently transfected with non‐target siRNA control (Control), siRNA targeting HCK (siHCK), and siRNA target GBP2 (siGBP2). mRNA and protein expressions of A,B) GBP2 and C,D) HCK in the transfected cells were examined by qPCR and immunofluorescence staining with anti‐GBP2 and anti‐HCK antibodies, respectively. Representative images of the transfected cells stained with anti‐HCK and anti‐GBP2 antibodies were shown. E) CCK8 assay to measure relative proliferation in the indicated groups. F) Ki67 immunofluorescence staining of the indicated groups. G) TUNEL assay to measure apoptosis in transfected cells. Mean apoptotic cell percentages are plotted. Data are represented as mean ± SEM. *P < 0.05, **P < 0.01. *P* < 0.05 is considered as statistically significant. Each experimental group contained 6 samples. Differences between group comparisons were evaluated using Kruskal‐Wallis Test. Adjustment by Bonferroni correction was used for multiple comparisons.

To characterize the roles of GBP2 and HCK in endometriosis, siRNA knockdowns of GBP2 (siGBP2) and HCK (siHCK) were conducted in human endometriotic stromal cells (hEMSCs). Both quantitative PCR (qPCR) analysis and immunofluorescence (IF) staining confirmed the silencing of GBP2 in siGBP2 cells (**Figure** **3**A,B) and the silencing of HCK in siHCK cells (Figure 3C,D). Functionally, HCK and GBP2 knockdown diminished the viability and proliferation of transfected stromal cells, as measured by the CCK8 assay (Figure 3E) and Ki67 staining (Figure 3F). In addition, apoptotic cell percentage increased roughly two‐fold following HCK or GBP2 knockdown (Figure 3G). These results suggest that endometriotic stromal cells rely on HCK and GBP2 function to survive, supporting the hypothesis that their pharmacological inhibition may be beneficial in treating endometriotic tissue.

**Figure 4:**
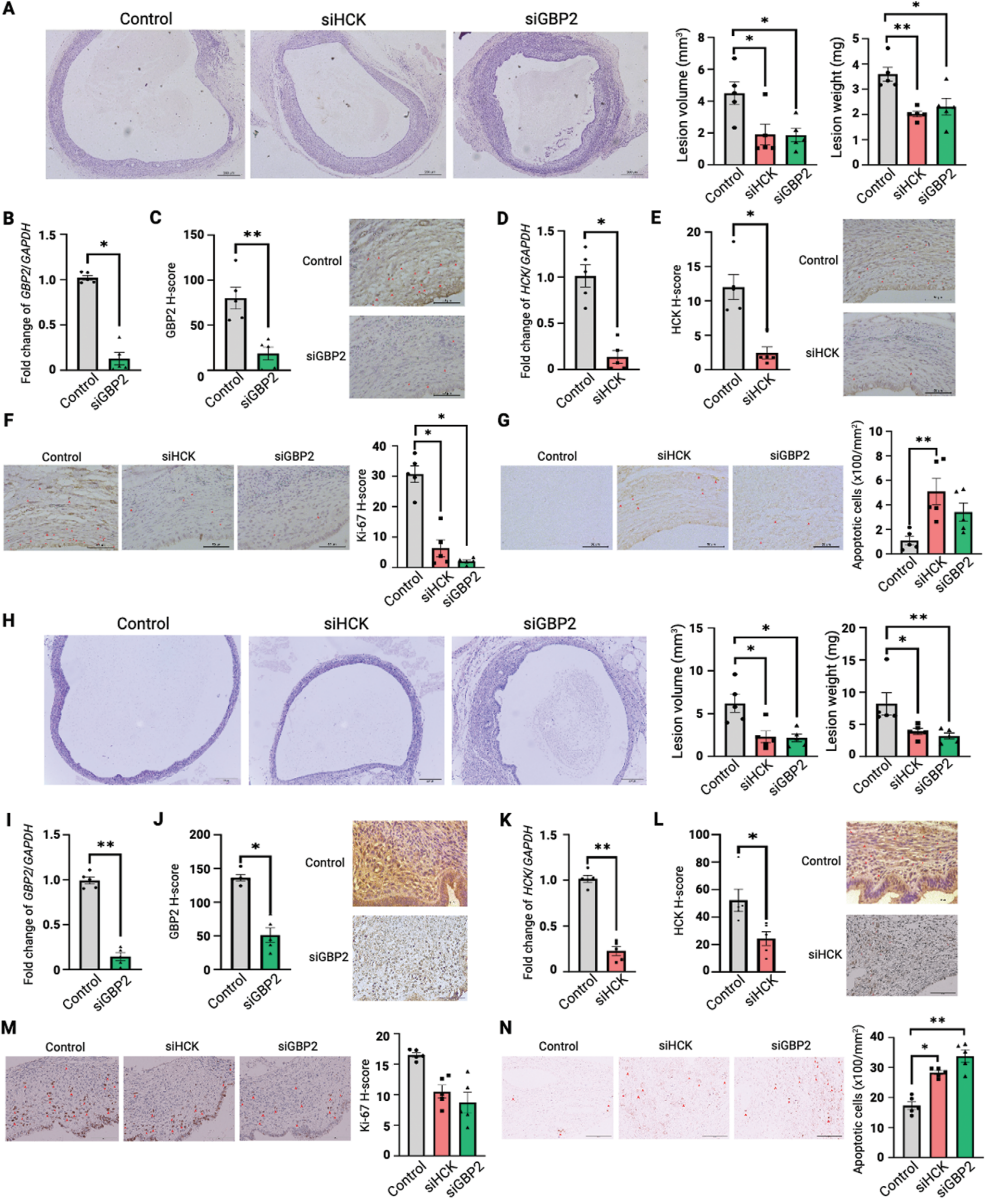
GBP2 and HCK knockdown independently suppressed endometriosis in vivo. The effect of knocking down GBP2 and HCK in endometriosis was assessed by both subcutaneous A–G) and intraperitoneal H–N) endometriosis mouse models (*n* = 5). A,H) The volume and weight of the excised lesions were recorded and compared. Representative images of the paraffin‐embedded murine ectopic endometrium xenografts stained with hematoxylin and eosin were shown. mRNA and protein expressions of (B‐C, I‐J) GBP2 and (D‐E, K‐L) HCK in murine endometriosis xenografts were analyzed by qPCR and IHC. Representative images of the paraffin‐embedded xenografts stained with anti‐GBP2 and anti‐HCK antibodies were shown. (F, M) Cell proliferation and (G, N) apoptosis status of the xenograft treated with siRNA against GBP2 and HCK were analyzed by Ki‐67 IHC staining and TUNEL assay, respectively. Representative images of each experimental group were displayed. Data are represented as mean ± SEM. *P < 0.05, **P < 0.01. *P* < 0.05 is considered as statistically significant. Differences between group comparisons were evaluated using Kruskal‐Wallis Test. Adjustment by Bonferroni correction was used for multiple comparisons.

To evaluate GBP2 and HCK function in vivo, we performed independent siRNA knockdown of both targets in subcutaneous and intraperitoneal endometriosis mouse models. Following implantation of endometrial tissue, the mice were subjected to daily siRNA treatments for a week (Figure S3A, Supporting Information). As reflected by the steady increase in body weight, subcutaneous treatments with siGBP2 and siHCK demonstrated no deleterious effect on the mice (Figure S3B, Supporting Information). In contrast to mice treated with control siRNA, the volume and weight of subcutaneous lesions in mice treated with siGBP2 and siHCK were significantly reduced (**Figure** **4**A). Animals treated with siGBP2 showed reduced GBP2 expression in lesions at mRNA (Figure 4B) and protein levels (Figure 4C). Similarly, *HCK* was successfully silenced by siHCK treatment (Figure 4D,E). Consistent with the in vitro results, siGBP2 and siHCK treatments decreased endometriotic cell proliferation as measured by Ki67 staining (Figure 4F). Furthermore, an increased proportion of cells underwent apoptosis following inhibitions of GBP2 and HCK as measured by TUNEL staining, although the effect of siGBP2 failed to reach statistical significance (Figure 4G). Similar to the subcutaneous treatment, intraperitoneal treatments of siGBP2 and siHCK significantly reduced ectopic lesion growth (Figure 4H). Target knockdown was confirmed for both siGBP2 and siHCK2 (Figure 4I–L). siGBP2 and siHCK treatments tended to decrease the proliferative ability of the endometriotic cells (Figure 4M) and significantly induced apoptosis in the ectopic lesions (Figure 4N). These collective data demonstrate that GBP2 and HCK inhibition independently attenuated endometriosis pathology through suppression of cell proliferation and activation of apoptosis, highlighting the potential of these therapeutic interventions for treating endometriosis.

### 2.4 Identification of ITGB2 as a Potential Therapeutic Target for Drug Repurposing

**Figure 5:**
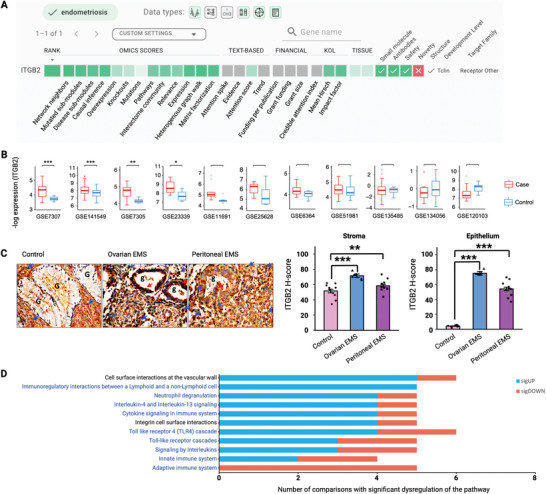
Identification of ITGB2 as a potential high‐confidence target for endometriosis. A) Screenshot of the Target ID page of PandaOmics for endometriosis meta‐analysis. ITGB2 was revealed as a druggable target with high confidence for endometriosis. B) The expressions of *ITGB2* in the eleven endometriosis‐related comparisons were displayed in box plots. *FDR < 0.05, **FDR < 0.01, ***FDR < 0.001. FDR < 0.05 indicates a significant differential expression. C) ITGB2 protein expression in the paraffin‐embedded human endometriosis and normal endometrium tissues were assessed by H‐score. Control (*n* = 10), ovarian EMS (*n* = 7), and peritoneal EMS (*n* = 10). Representative images of the human tissues stained with anti‐ITGB2 antibody were displayed. G) endometrium glands; g: endometriotic glands; blue arrows, positive staining in endometrial or endometriotic stromal cells; red arrows, positive staining in endometrial or endometriotic epithelial cells. Magnifications, 40x. Data are represented as mean ± SEM. *P < 0.05, **P < 0.01, ***P < 0.001. *P* < 0.05 is considered as statistically significant. D) Dysregulated pathways associated with *ITGB2* in endometriosis comparisons were displayed. Pathways labeled in blue are immune‐associated. Differences between group comparisons were evaluated using Kruskal‐Wallis Test. Adjustment by Bonferroni correction was used for multiple comparisons.

In parallel to our efforts to identify novel targets for treating endometriosis, we investigated targets with approved compounds that could be repurposed. Consequently, we identified integrin subunit beta 2 (ITGB2) as a potential druggable target for endometriosis. ITGB2 ranked as the 1^st^ target in the meta‐analysis under a high confidence setting (Figure S4, Supporting Information) and scored ≥0.8 in 7 out of the 13 Omics models (**Figure** **5**A). ITGB2 was not regarded as an essential gene in the Database of Essential Genes and is targeted by FDA‐approved drugs, so it was considered a safe target in PandaOmics’ evaluation. ITGB2 was upregulated in 8 out of 11 (72.7%) bulk transcriptomic comparisons in human ectopic endometrial lesion samples relative to controls (Table S4, Supporting Information) and showed statistical significance in 4 of these comparisons (Figure 5B; and Table S4, Supporting Information). IHC analysis in paraffin‐embedded human EMS tissue samples revealed a significant elevation in ITGB2 expression in stromal cells and epithelial cells within ovarian EMS and peritoneal EMS compared to normal endometrial stromal and epithelial cells (Figure 5C). In addition to the expression dysregulation analysis, pathway enrichment analysis was conducted to explore the role of ITGB2 in endometriosis. Nine altered pathways linked with ITGB2 were involved in the immune response (Figure 5D; and Table S5, Supporting Information), with 7 of them primarily upregulated, indicating that ITGB2 could be associated with immune dysregulation in endometriosis.

### 2.5 Inhibition of ITGB2 by Lifitegrast Inhibited Endometriotic Growth

As ITGB2 was upregulated in human ectopic endometrial tissues (Figure 5B), we hypothesized that Lifitegrast, an antagonist of lymphocyte function‐associated antigen 1 (LFA‐1) that is approved for dry eye disease, may be a candidate intervention for treating endometriosis. Integrin LFA‐1 is an ITGB2/ITGAL heterodimer expressed on the surface of T cells to modulate their activation and migration.^[^ ^36^ ^]^ During inflammation, ICAM1, which is expressed on the surface of mature antigen‐presenting cells, damaged epithelium, or endothelium, binds to LFA‐1 to trigger cytokine release.^[^ ^37^ ^]^ Lifitegrast competes with ICAM1 for LFA‐1 to inhibit this interaction and thus suppress inflammation (Figure S5A, Supporting Information). In line with this known function, ICAM1 expression was analyzed and found to be overexpressed in 8 out of 11 (72.7%) omics datasets, with five instances of overexpression having FDR < 0.05 (Figure S5B and Table S4, Supporting Information).

**Figure 6:**
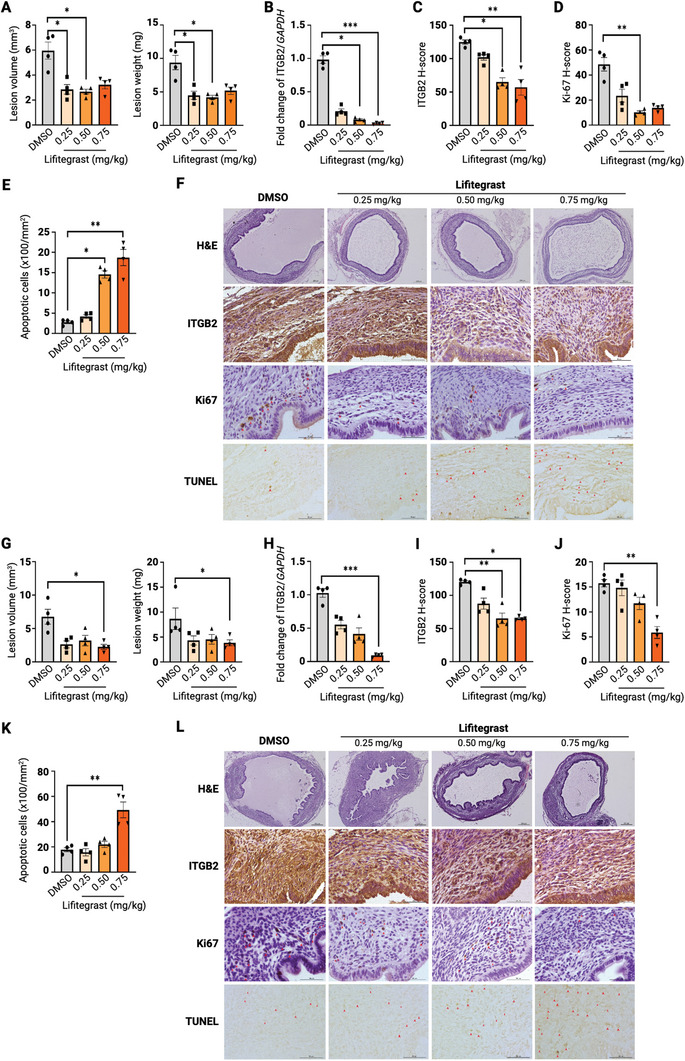
Lifitegrast inhibited murine endometriotic growth. The efficacy of lifitegrast in treating endometriosis was determined by subcutaneous A–F) and intraperitoneal G–L) administration of lifitegrast in endometriosis mouse models (*n* = 4). A,G) The volume and weight of the excised lesions treated with DMSO or Lifitegrast at the indicated doses were recorded and compared. B,H) mRNA and C,I) protein expressions of ITGB2 in the murine ectopic endometrium xenograft were analyzed by qPCR and IHC. D,J) Cell proliferation and E,K) apoptosis status of the xenograft were analyzed by Ki‐67 IHC staining and TUNEL assay, respectively. F,L) Representative images of paraffin‐embedded murine ectopic endometrium xenografts stained with hematoxylin and eosin, anti‐ITGB2 antibody, Ki‐67 IHC staining, and TUNEL assay in each experimental group were displayed. Data are represented as mean ± SEM. *P < 0.05, **P < 0.01, ***P < 0.001. *P* < 0.05 is considered as statistically significant. Differences between group comparisons were evaluated using Kruskal‐Wallis Test. Adjustment by Bonferroni correction was used for multiple comparisons.

To assess the efficacy of Lifitegrast in treating endometriosis, Lifitegrast was administered subcutaneously at three different doses (0.25, 0.50, and 0.75 mg kg^−1^) in the endometriosis mouse model (**Figure** **6**; Figure S5C, Supporting Information). In contrast to control DMSO treatment, Lifitegrast at 0.25 and 0.50 mg kg^−1^ significantly decreased the volume and weight of endometriotic lesions in mice (Figure 6A,F *first row*). Treatment with medium or high dose Lifitegrast (0.50 or 0.75 mg kg^−1^, respectively) significantly reduced ITGB2 mRNA and protein (Figure 6B,C,F *second row*). Functionally, treatment with 0.50 or 0.75 mg kg^−1^ Lifitegrast significantly suppressed endometriotic cell proliferation (Figure 6D,F *third row*) and increased the apoptotic cell number (Figure 6E,F *fourth row*) in the lesion as compared to control lesions. Intraperitoneal administration of Lifitegrast was also evaluated similarly in separate experiments (Figure 6G–L). Lifitegrast treatment at 0.25 and 0.75 mg kg^−1^ significantly reduced ectopic lesion growth (Figure 6G,L *first row*). Lifitegrast treatment of 0.75 mg kg^−1^ reduced mRNA and protein expressions of ITGB2, (Figure 6H,I,L *second row*), suppressed endometriotic cell proliferation (Figure 6J,L *third row*), and increased apoptosis (Figure 6K,L *fourth row*). Together, these results demonstrate the therapeutic efficacy of Lifitegrast in treating endometriosis in an ITGB2‐dependent manner.

## 3 Discussion

Here, we used an AI‐based target discovery platform to uncover novel targets for endometriosis, a disease without a cure that affects millions of women globally.^[^ ^27^ ^]^ This approach yielded two major discoveries: i. We identified GBP2 and HCK as previously unappreciated therapeutic targets for endometriosis ii. Lifitegrast, a clinically approved ITGB2 inhibitor, may be repurposed as an endometriosis treatment. Notably, these discoveries were made within one year, highlighting the extraordinary speed of AI‐aided drug and target discovery. Recently, AI has made excellent achievements in accelerating the tedious target discovery for drug development.^[^ ^9^ ^]^ Generative adversarial networks, one of the key concepts in modern AI, successfully demonstrated their effectiveness in hastening target identification.^[^ ^38^, ^39^, ^40^ ^]^ PandaOmics incorporates over 20 time‐machine‐validated AI and bioinformatics models generated from multi‐omics and text data, such as publications, grants, and patents, for therapeutic target exploration. It allows the disclosure of hidden hypotheses unreported by existing knowledge or simple bioinformatics analysis and helps to define druggable targets in a simple manner and faster.^[^ ^10^, ^11^, ^35^, ^41^, ^42^ ^]^ This study provides an additional piece of evidence for the effectiveness of AI in deriving therapeutic targets and potential repurposing candidates.

Following the AI‐aided identification of two novel targets, GBP2 and HCK, we evaluated their expression patterns in endometriotic tissue in which we observed their upregulation in multiple datasets (Figure 2). In agreement, we observed their elevated expression in hEMSC (Figure 3) and endometriosis mouse models (Figure 4). Knockdowns of GBP2 and HCK offered therapeutic effects on endometriosis by reducing proliferation and increasing apoptosis of the endometriotic cells. With no previous study in endometriosis and scarce publications in other immune indications,^[^ ^43^ ^]^ GBP2 emerged as a novel target in endometriosis. Upon IFN‐γ exposure, it is one of the two proteins induced in cells or tissue among the seven members of the GBP family.^[^ ^44^ ^]^ GBP2 is primarily involved in host defense,^[^ ^45^, ^46^ ^]^ as supported by findings of loss‐of‐function analysis in mice.^[^ ^47^, ^48^ ^]^ Specifically, GBP2 activates caspase‐dependent apoptosis and controls macrophage apoptosis and pyroptosis. A recent diabetic nephropathy study identified GBP2 as a positive modulator of M1 polarization by activating NOTCH1 signaling to drive inflammation.^[^ ^49^ ^]^ Macrophages are pivotal players in endometriosis, acting as double‐edged swords.^[^ ^50^ ^]^ Endometriotic lesion‐resident macrophages exhibit origin‐dependent functions: endometrial macrophages promote endometriosis, while newly recruited monocyte‐derived macrophages suppress lesion development.^[^ ^51^ ^]^ Various studies have also demonstrated a positive correlation between macrophages and endometriosis‐associated pain, as macrophages are densely populated in regions with severe nerve infiltration.^[^ ^52^, ^53^ ^]^ This regional nerve infiltration pattern may explain the elevated pain scores in endometriosis patients.^[^ ^54^, ^55^, ^56^ ^]^ Although the dysregulated pathway results (Figure 2E) provided minimal cues about the immune role of GBP2, they aligned with the literature‐derived functions of GBP2 in other disease settings. Thus, these collective results support the possible role of GBP2 in inducing immune dysregulation during endometriosis.

In contrast to GBP2, HCK has an established role in the immune response and HCK activation is implicated in antiviral immunity^[^ ^57^, ^58^, ^59^ ^]^ and inflammation.^[^ ^60^, ^61^ ^]^ In cancer, HCK promotes macrophage polarization towards the tumor‐promoting M2 phenotype, enhances the release of pro‐inflammatory cytokines, and promotes the infiltration of myeloid‐derived suppressor cells.^[^ ^62^, ^63^ ^]^ Downregulation of HCK in endometriosis induces a protective effect by decreasing the cytotoxicity of natural killer cells in the ectopic lesion microenvironment.^[^ ^64^ ^]^ Our findings are consistent with the literature (Figures 3 and 4), as indicated by the upregulation of HCK‐associated immune pathways and the protective role of HCK inhibition in endometriosis.

Our study advances the understanding of endometriosis pathogenesis by providing new insights into the complex immune dysregulation underlying the development and progression of this condition. Identifying GBP2 and HCK as novel therapeutic targets highlights that specific immune‐mediated mechanisms drive endometriosis, bringing our attention to the immunological aspects of the disease and paving the way for more targeted, effective treatment strategies. That GBP2 and HCK knockdown exhibit therapeutic effects in preclinical models underscores their potential for future drug development. Modulating GBP2 and HCK‐mediated processes, such as macrophage polarization, caspase‐dependent apoptosis, and natural killer cell function, may represent a complementary approach to existing endometriosis treatments, which primarily focus on hormonal or surgical strategies. This diversification of treatment options could benefit the personalized management of endometriosis, where therapies can be tailored to the specific immune profiles and dysregulated pathways observed in individual patients.

Our target identification analysis also nominated ITGB2 as another unappreciated target in endometriosis pathology, which has been implicated in multiple immune functions.^[^ ^37^, ^65^, ^66^, ^67^ ^]^ The clinically approved drug Lifitegrast, which potently inhibits ITGB2 function, was proposed for treating endometriosis. ITGB2 can bind with ITGAL, ITGAM, ITGAX, or ITGAD to form different phenotypes of β2 integrins based on various immune cell types.^[^ ^68^ ^]^ The ITGAL/ITGB2 heterodimer, denoted as LFA‐1, is expressed in all lymphocytes and leukocytes and is particularly important for T cell function and inflammation.^[^ ^36^ ^]^ In endometriosis, we first observed the overexpression of ITGB2 in patients, as evident by the results from both transcriptomic datasets and IHC staining (Figure 5). Notably, Lifitegrast suppressed ectopic lesion growth in a murine endometriosis model (Figure 6, Figure S5, Supporting Information). Considering the mild treatment‐emergent adverse events in patients with dry eye disease,^[^ ^69^ ^]^ our findings suggest that its utilization in endometriosis treatment may be well‐tolerated in patients. This hypothesis warrants further investigation to validate its clinical significance and translational value.

In conclusion, this study uncovers the roles of two AI‐derived novel druggable targets, GBP2 and HCK, in endometriosis, whose inhibition delayed disease progression in multiple preclinical models. Lifitegrast, an antagonist of ITGB2, demonstrated its efficacy in alleviating endometriotic lesion growth, indicating its potential to be repurposed for endometriosis. There are some limitations to the current study. The expression of GBP2, HCK, and ITGB2 in matched eutopic endometrium and endometriosis lesions was not explored due to sample constraints. The effects of GBP2 and HCK inhibition on normal endometrial cells in vitro and on fertility and pregnancy outcomes in vivo need to be addressed to validate the absence of side effects of the treatments. Future studies are needed to fully elucidate the underlying mechanisms of their actions and evaluate the clinical efficacy of interventions targeting these molecules for the treatment of endometriosis.

## 4 Experimental Section

### Data Source and Availability

A total of thirty‐six endometriosis‐related bulk transcriptomic datasets, comprising microarray and RNA‐sequencing series, of various tissue sources retrieved from Gene Expression Omnibus (GEO) and ArrayExpress were available for direct downstream analysis and target identification on PandaOmics.

### Dataset and Comparison Selection

Datasets with ectopic endometrial tissue were selected for target identification. Based on the availability of information on disease subtype and endometrial cycle phase, twenty case‐control comparisons were generated from thirteen bulk transcriptomic datasets, which were listed in Table S1 (Supporting Information). Both normal endometrial tissue from healthy individuals and eutopic endometrial tissue from patients with endometriosis were chosen as the controls, which was labeled as “Healthy” and “Eutopic endometrium”, respectively.

### Meta‐Analysis

To identify potential targets for endometriosis, eleven subtype and phase unspecified case‐control bulk transcriptomic comparisons were pooled into a meta‐analysis for therapeutic target exploration (Table S1, Supporting Information).

### Target Identification by PandaOmics

PandaOmics was a cloud‐based target discovery platform with multiple deep learning models and AI algorithms incorporated in the target prioritization process.^[^ ^70^ ^]^ The platform employed a diverse range of computational approaches that began with multiomics datasets derived from tissue samples of patients with endometriosis, serving as a basis for the target discovery process (Figure 1, *step 1*). In addition to these experimental data, the platform incorporated biological network analysis and text‐mining methods to generate target hypotheses that were grounded in both omics data and prior evidence from scientific literature, clinical trials, and grant applications (Figure 1, *step 2* and Figure 2A). Specifically, the pipeline combined several distinct but complementary computational approaches, including random walk on heterogeneous graphs and negative matrix factorization, to produce a ranked list of target genes (Video S1, Supporting Information). These AI‐driven methods were able to capture trends and even make projections into the future (Figure 2A; Figure S2, Supporting Information). A total of twenty‐three target prioritization models covering Omics, Text‐based, Financial, and Key Opinion Leader (KOL) data were developed to predict the association between the target genes and the endometriosis indication. Scores of each of the models are given in a normalized scale from zero to one, with higher scores corresponding to better target‐disease association as predicted by the model. These models were validated using a Time Machine approach to confirm their abilities in target identification and were equally weighted in the calculation of the overall rank of each target. This ensemble approach helped improve the reliability and reproducibility of the target identification process. Furthermore, adjustable filters for druggability, tissue specificity, target family, and developmental status were also available to refine the target list. The druggability of a target is comprehensively assessed through three critical dimensions: small molecule accessibility, safety, and novelty. Small molecule accessibility is defined by the inclusion of the gene within recognized druggable families and the presence of known small molecules targeting the gene's product. This data was meticulously gathered from the Open Targets platform, ClinicalTrials.gov, and the Target Central Resource Database. Safety was evaluated based on the gene's essentiality and the targeting of the gene's product by small molecules within clinical trial settings, with pertinent data sourced from the Database of Essential Genes and ClinicalTrials.gov. Novelty was determined through an analysis of the volume of related scientific publications, as systematically quantified by a proprietary AI engine. This engine assesses the level of scientific interest and the breadth of existing research on the gene. Definitions and data sources of the scores and filters were described in PandaOmics’ User Manual (https://insilico.com/pandaomics/help) and published by Kamya et al.^[^ ^57^ ^]^ This platform was available on a software‐as‐a‐service basis at https://pandaomics.com.

To identify actionable targets with various novelty levels, settings of filters and scores were customized to reprioritize the targets. Targets belonging to the druggable protein classes, and not regarded as essential genes defined by the Database of Essential Genes were retained in the analysis. In order to identify targets for antagonist development, genes that were detected in over 50% of case‐control comparisons and showed consistent upregulation across those comparisons were selected, yielding 1, 656 genes for target prioritization (Table S6, Supporting Information). As a result, two lists (Tables S2 and S3, Supporting Information) of druggable targets with high confidence and novelty were generated with the filters and scores set according to Figures S2 and S4 (Supporting Information) in PandaOmics, and their top 50 targets were subjected to further analysis. Targets with high confidence were defined by being well‐studied in indications other than endometriosis and having approved drugs, while novel targets referred to targets with scarce knowledge in their biological functions or disease pathogenesis.

### Pathway Analysis

Signaling pathway activation status was assessed using proprietary single network model iPANDA.^[^ ^71^ ^]^ With the combinational use of differential gene expression data and the degree of pathway topology decomposition, iPANDA robustly identified sets of biological relevant pathway signatures from the input data with significant noise reduction. iPANDA value of 1 and ‐1 indicated pathway activation and suppression, respectively. Hierarchical organization of signaling pathways were curated based on the Reactome database.

### Clinical Endometriotic Samples

The sections of clinical tissue sample were collected from biobank of Department of Obstetrics and Gynaecology, CUHK, including normal endometrial tissue (*n* = 10), ovarian endometriotic tissue (*n* = 10) and peritoneal endometriotic tissue (*n* = 10)

### Clinical Endometriotic Samples—Inclusion Criteria for Clinical Study and Sampling

1. Complaints of pelvic pain, dysmenorrhoea and/or dyspareunia; and
2. Surgical and pathology confirmed endometrioma; and
3. Undergoing surgical treatment.

### Clinical Endometriotic Samples—Exclusion Criteria for Clinical Study and Sampling

1. Secondary dysmenorrhoea due to gynecological conditions other than endometriosis, e.g. pelvic inflammatory diseases, genitourinary infections, gynecological tumors, etc; or
2. Primary dysmenorrhoea without any underlying disease identified; or
3. Ultrasound suggested hemorrhagic ovarian cyst, ovarian dermoid cyst, cystic neoplasm, tubo‐ovarian abscess or other ovarian pathology; or
4. Ultrasound confirmed adenomyosis; or
5. Endometriosis under active medication in the past 1 month.

### Cell Line and Cell Culture

hEMSCs (Cell line HS293 (C).T) were purchased from ATCC (Manassas, VA, USA), and only cells from passages 4–6 were used in this study. The hEMSCs were seeded in a 24‐well culture dish and transfected with human HCK siRNA (Horizon Discovery, L‐003141‐00‐0005, 10 nm), human GBP2 siRNA (Horizon Discovery, L‐011867‐00‐0005, 10 nm) or non‐targeting control siRNA (Horizon Discovery, D‐001810‐10‐05, 10 nm) according to the manufacturer's instructions (*n* = 6 per group).

### Mouse Model of Endometriosis

All animal works were approved by the Animal Experimentation Ethics Committee of the Chinese University of Hong Kong and performed under ethics 21‐139‐MIS in accordance with international standards and guidelines. 7‐week‐old C57BL/6 female mice provided by the Laboratory Animal Service Center were housed in a pathogen‐free animal house with chow and tap water.

Two kinds of endometriosis models were established by subcutaneous or intraperitoneal transplantation. To synchronize the estrous cycle in each mouse, ovariectomy was performed 7 days before transplantation. 100 µg kg^−1^ estradiol‐17β (E_2_; Sigma, E2758) were intramuscularly injected at the time of ovariectomy and on every 5 days to synchronize the E2 level. Mice in the donor group were sacrificed to obtain the endometrial tissue of the uterine fragments, not including myometrial tissue, which was then cut into endometrial fragments of 2 mm in diameter using a dermal biopsy punch (Miltex). In the subcutaneous model, a 3 mm skin incision on the abdominal wall of each recipient mice was made, thus subcutaneous pockets were created. Three endometrial tissues were placed into the pocket and then the skin incision was closed with 5‐0 suture (Johnson & Johnson, USA, W500H) and disinfected with iodine, then mice were kept on a warm pad for recovery. And the intraperitoneal endometriosis model was established by surgical transplantation of syngeneic endometrial tissues to the intestinal mesentery. Four endometrial fragments were carefully sutured to intestinal vessels ≈0.5 cm from the bowel with 7‐0 sutures (Johnson & Johnson, USA, J488G). The method of suture rather than injection transplantation was used to ensure the growth of the lesions in a designated location in the peritoneal cavity, facilitating the evaluation of the therapeutic intervention. The abdominal wall and skin were closed with 5‐0 suture and disinfected with iodine.

For siRNA treatment, the mice were then randomly assigned to one of the following treatments (*n* = 5 per group): non‐targeting control siRNA as the negative control, mouse HCK siRNA (Horizon Discovery, L‐040986‐00‐0005), mouse GBP2 siRNA (Horizon Discovery, L‐040199‐00‐0005, 10 nm) by subcutaneous injected to lesion by micro‐syringe on subcutaneous model or by intraperitoneal injection on intraperitoneal model, every day for 1 week started from 2 weeks after the surgery until termination for lesions collection (Figure S3A, Supporting Information). siRNA would be incorporated into DOPC at a ratio of 1:10 (w/w) siRNA/DOPC. Tween 20 would be added to the mixture in a ratio of 1:19 Tween 20: siRNA/DOPC. The mixture would be vortexed, frozen in an acetone/dry ice bath, and lyophilized. Before in vivo administration, this preparation would be hydrated with normal 0.9% saline to achieve the desired dose in 150 to 200 µL per injection.^[^ ^72^ ^]^ Each experimental group consisted of 5 mice.

For medical treatment, subcutaneous and intraperitoneal injections of the vehicle as a negative control (DMSO) and administration of Lifitegrast (Cayman, 22 588) at 0.25, 0.50, and 0.75 mg kg^−1^ were performed every day for 1 week, starting from 2 weeks after the surgery until termination for lesions collection, as in siRNA treatment. Each experimental group consisted of 4 mice.

The conditions of the mice were checked daily, and body weights were monitored regularly during the experiment. At the end of treatment, to assess the effects in inhibiting lesion growth, volume and weight of the endometriotic lesions were measured. Lesion volume (mm^3^) was determined using the formula, length (mm) × width^2^ (mm^2^) × ½.

### Quantitative RT‐PCR

RNA was extracted by RNAiso Plus (Takara, 9108) according to the manufacturer's protocol. Concentration and quality of RNA were measured by Nanodrop spectrophotometry (Wilmington) and reverse‐transcribed with PrimeScript RT Master Mix (TaKaRa, RR036A) according to the manufacturer's protocol. TB Green Premix Ex Taq (Tli RNaseH Plus) (Takara, RR420A) was used to amplify the expressed genes and quantified by LightCycler 480 quantitative PCR system (Roche, Switzerland). *GAPDH* gene was used as the house‐keeping gene to normalize the expression of target genes. PCR primers are listed in the Table S7 (Supporting Information).

### Immunofluorescence Staining

hEMSCs were grown on glass coverslips. After the treatment, hEMSCs were fixed with 4% paraformaldehyde and permeabilized with 0.5% Triton X‐100. After protein block to reduce non‐specific signal, the fixed cells were incubated with primary antibodies against HCK (Thermo Scientific, MA5‐15371, 1:200), GBP2 (Abcam, ab203238, 1:200), and Ki67 (Abcam, ab16667, 1:200) at room temperature for 1 h. This procedure was followed by incubation with secondary antibodies Alexa Fluor 488 (Thermo Scientific, A11034, 1:1000) and Alexa Fluor Plus 594 (Thermo Scientific, A32742, 1:1000) for 1 h. Slides mounted with coverslips were viewed under a fluorescence microscope (Olympus). Images were obtained at ×400 magnification using a digital camera under the same light conditions and exposure times. For semi‐quantitative analysis, protein expressions were quantified by H‐score. The scores were generated blindly and independently by two authors (Y Lin and CC Wang) by Qupath.

### Hematoxylin and Eosin Staining and Immunohistochemistry

The endometriotic lesion from the endometriotic mouse model was fixed in 10% buffered formalin for 24 h and embedded in paraffin blocks, cut into slices of 3 µm thickness. Hematoxylin and eosin staining was carried out in every 10^th^ section to confirm the microscopic structures in the lesions.

For IHC staining, sections from human tissue and mouse models were deparaffinized, incubated with 3% H_2_O_2_ in the dark to block the endogenous peroxidase activity. After antigen retrieval and protein block to reduce non‐specific signal, the sections were then incubated with a primary antibodies of HCK (Thermo Scientific, MA5‐15371 for human and PA5‐102573 for mouse, 1:200), GBP2 (Abcam, ab203238, 1:200), ITGB2 (Thermo Scientific, PA5‐116962, 1:200), and Ki67 (Abcam, ab16667, 1:200) overnight, followed by secondary antibody Goat anti‐Rabbit IgG H&L (Abcam, ab6721, 1:200) for 1 h and developed in 3,3‐diaminobenzidine (DAB) for 3–5 min. After mounting, Leica DM6000B microscope was used for image capturing. For semi‐quantitative analysis, protein expressions were quantified by H‐score. The scores were generated blindly and independently by two authors (Y Lin and CC Wang) by Qupath (control (*n* = 10), ovarian EMS (*n* = 7), and peritoneal EMS (*n* = 10)).

### CCK8 Assay

Cell proliferation of the transfected stromal cell line was evaluated by Cell Counting Kit‐8 (ApexBio, K1018). A density of 1000 cells per well was seeded in a 96‐well plate and three parallel wells were set for each group. At 0, 24, and 48 h after treatment, 10 µL CCK‐8 reagent and 100 µL DMEM medium were added. Then, the cells were incubated for 1 h at 37 °C and absorbance was measured at 450 nm.

### TUNEL Apoptosis Assay

Percentages of apoptotic cells in the transfected stromal cell population and murine lesions were determined by DeadEnd Colorimetric TUNEL System (Promega, G7130) based on the manufacturer's protocol.

### Statistical Analysis

Statistical analyses were performed by SPSS software version 25.0 (SPSS Science, Chicago, USA). Differences between groups were evaluated by One Way ANOVA if data distribution normal or Kruskal–Wallis test if data distribution skewed. Differential expression analyses were conducted using the limma method. Adjustment by Bonferroni correction was used for multiple comparisons. *P* ≤ 0.05 was considered statistically significant.

### Ethical Statement

All animal experiments reported in this study were conducted in strict accordance with international standards and guidelines. The Animal Experimentation Ethics Committee of the Chinese University of Hong Kong approved all procedures involving animals under the ethics approval number 21‐139‐MIS.

## Conflict of Interest

B.H.M.L., X.L., A.G., F.R., A.Z., and F.W.P. are affiliated with Insilico Medicine, a commercial company developing AI solutions for aging research, drug discovery, and longevity medicine. Y.L., S.W.H., and C.C.W. declare no competing interests.

## Author Contributions

B.H.M.L., Y.L., and X.L. contributed equally to this work. B.H.M.L., Y.L., and S.W.H. conducted data investigation. X.L. and A.G. performed data curation. B.H.M.L., Y.L., X.L., S.W.H., F.W.P., and C.C.W. performed data analysis. B.H.M.L. and Y.L. worked on data visualization. F.R., A.Z., and C.C.W. provided methodology. F.R., F.W.P., and C.C.W. provided supervision. F.W.P. conducted project administration. A.Z. and C.C.W. offered conceptualization. C.C.W. performed Funding acquisition. B.H.M.L., Y.L., and X.L. wrote the original manuscript. A.G., F.W.P., and C.C.W. reviewed and edited the manuscript.

## Supporting information



Supporting Information

Supplemental Tables

Supplemental Video 1

## Data Availability

The data that support the findings of this study are available from the corresponding author upon reasonable request.
