# Supplementary material for: Utilizing AI for the Identification and Validation of Novel Therapeutic Targets and Repurposed Drugs for Endometriosis
Source: Adv Sci (Weinh). 2024 Dec 12;12(5):2406565. doi: 10.1002/advs.202406565 (PMC11792045; doi:10.1002/advs.202406565)
Supplement: Supplementary file 1 — Supporting Information [file ADVS-12-2406565-s002.docx]

**Supporting Information**

**Utilizing AI for the Identification and Validation of Novel Therapeutic Targets and Repurposed Drugs for Endometriosis**

*Bonnie Hei Man Liu, Yuezhen Lin, Xi Long, Sze Wan Hung, Anna Gaponova, Feng Ren, Alex Zhavoronkov, Frank W. Pun*, Chi Chiu Wang**


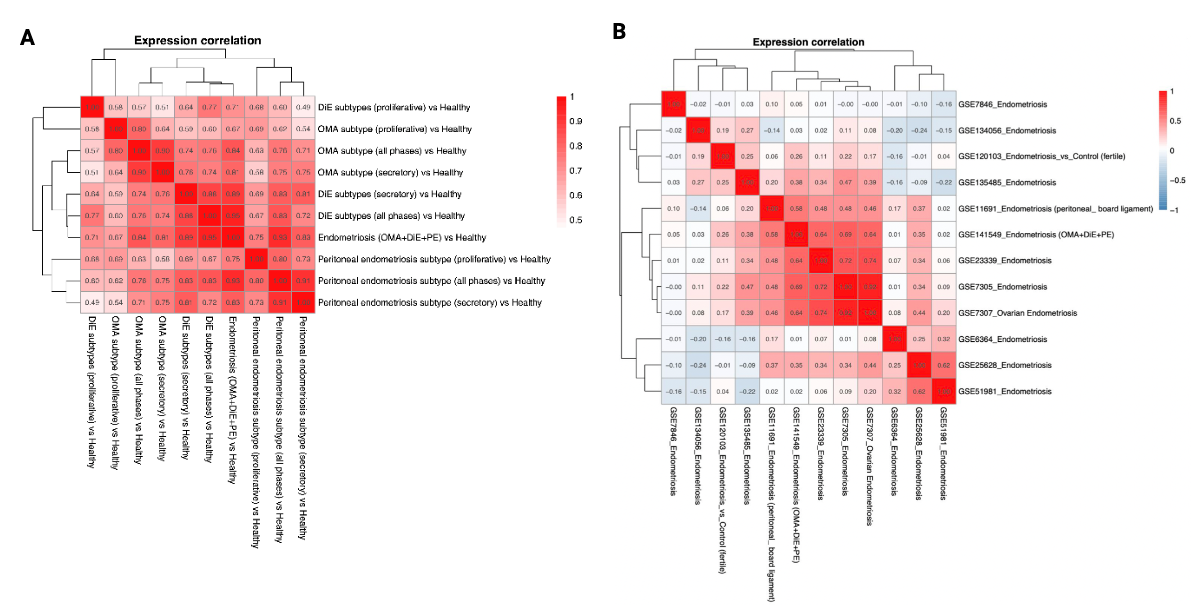


**Figure S1. Expression correlation of comparisons created from endometriosis transcriptomic datasets.** (A) Spearman’s correlation of the differential expression profiles (log fold-change values) of ten case-control comparisons created from GSE141549. Patient samples from GSE141549 were firstly divided based on their disease subtypes into the Peritoneal, DiE and OMA subtypes, yielding 3 subtype-specific comparisons. Then, the three subtypes of patients were further divided into the secretory and the proliferative uterine cycles, yielding another 6 uterine cycle-specific comparisons. Additionally, an overall subtype- and uterine cycle-nonspecific comparison between all patients and all controls was generated, resulting in a total of ten comparisons. In each comparison, patient samples were compared to their subtype- and cycle-matching controls. In total, 18,806 genes were detected, and their logarithmic fold changes (LFC) values retrieved from PandaOmics were subjected to Spearman's correlation analysis. (B) Expression correlation in 12 subtype- and uterine cycle-nonspecific comparisons derived from 12 datasets were also analyzed using Spearman's correlation. The degree of expression correlation is indicated by the red (positive)-blue (negative) color scale.


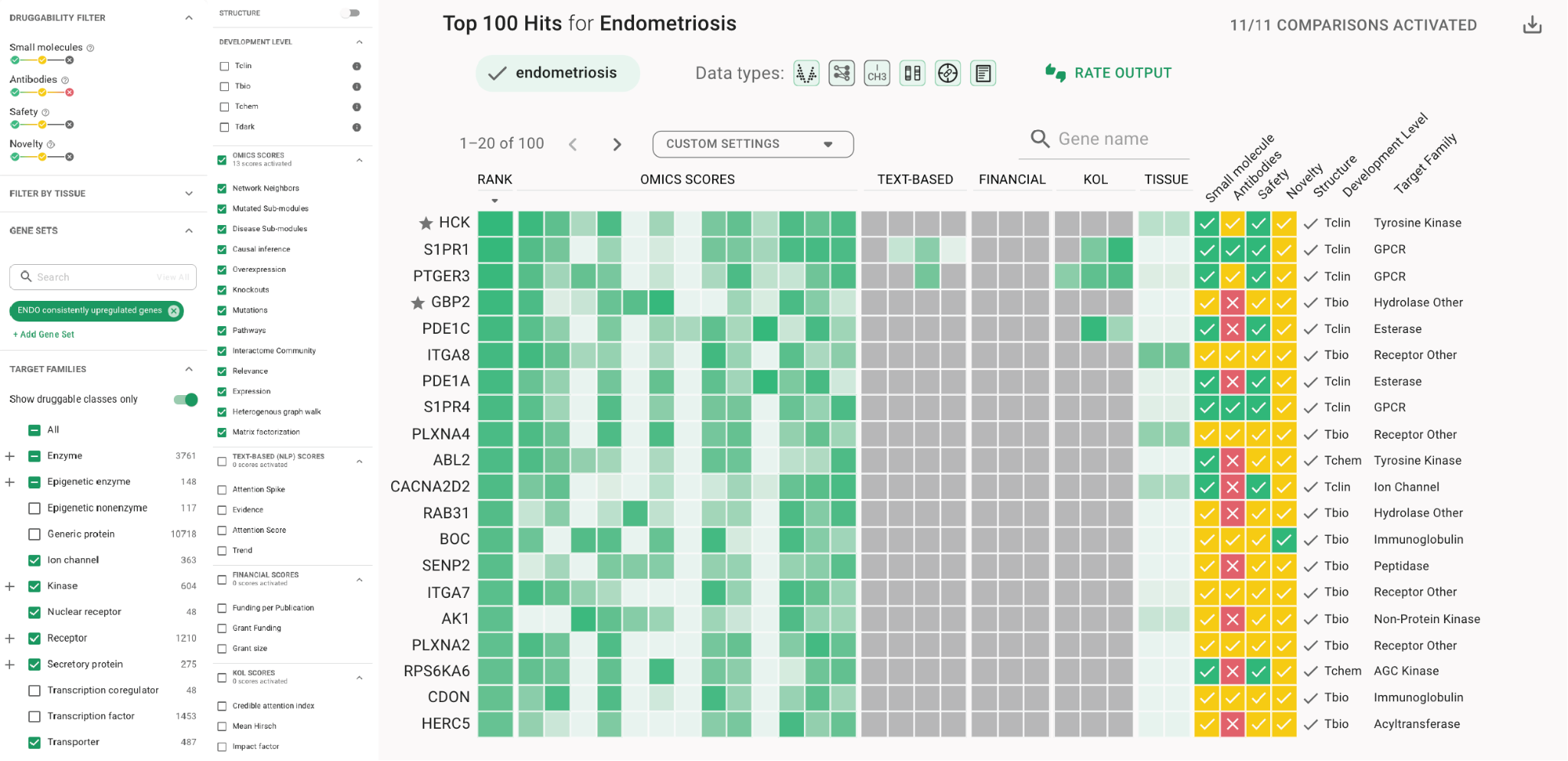


**Figure S2. Filter setting for novel target identification.**


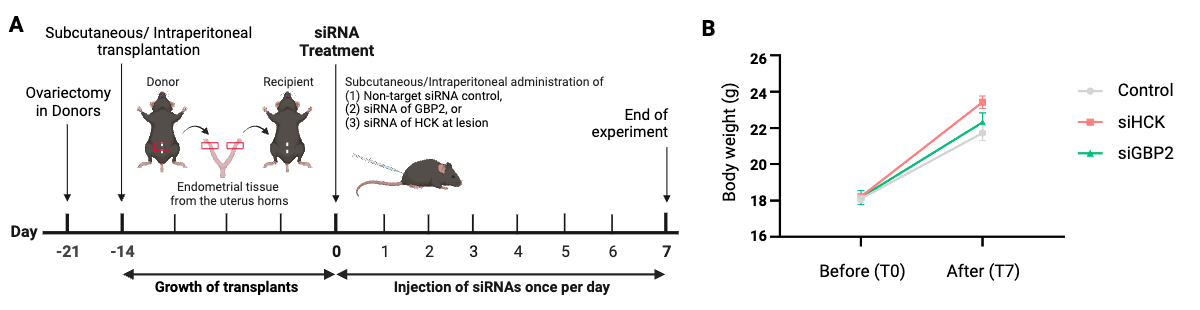


**Figure S3.** **In vivo siRNA knockdown endometriosis mouse model design and safety measurement.** (A) Timeline illustrating the establishment of the in vivo endometriosis model and siRNA treatment for investigating the effect of GBP2 and HCK. (B) To assess the safety of the siRNA treatment, body weights of the mice were measured before (D0) and after (D7) the treatment.


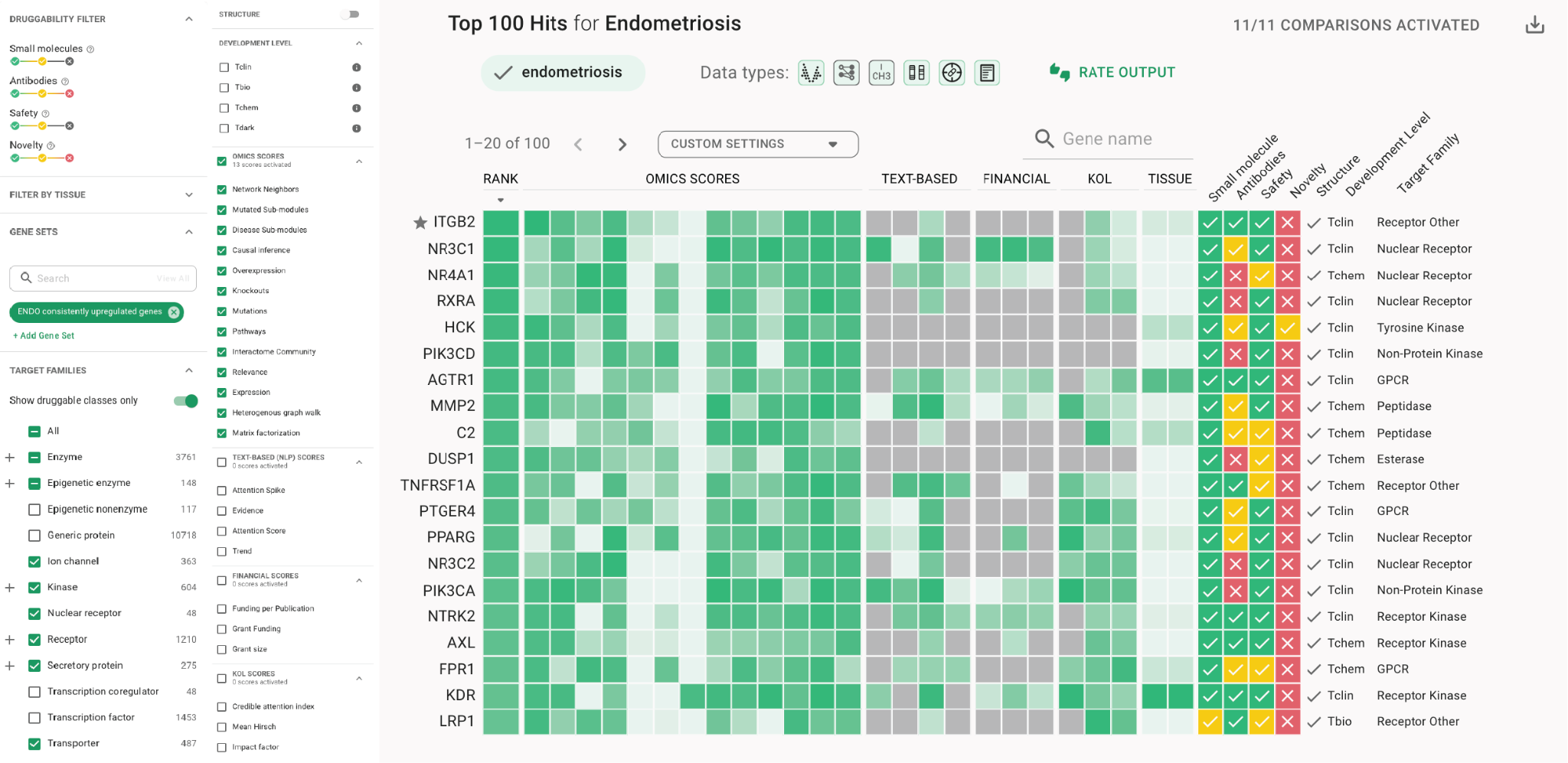


**Figure S4. Filter setting for high-confidence target identification.**


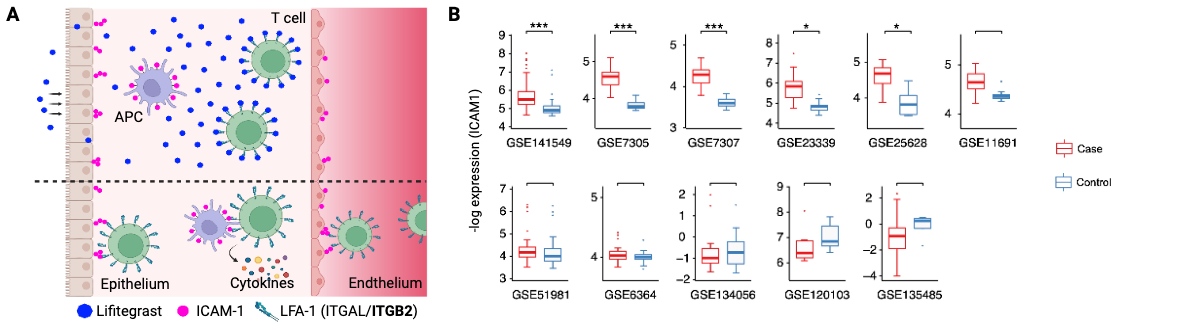


**Figure S5. Illustration on the mechanism of action of Lifitegrast.** (A) An illustration describing the mechanism of action of Lifitegrast. (B) The expressions of *ICAM1* in the eleven endometriosis-related comparisons were displayed in box plots. *FDR < 0.05, **FDR < 0.01, ***FDR < 0.001. FDR < 0.05 indicates a significant differential expression.

**Table S1.** Endometriosis case-control comparisons and their corresponding meta-analyses.

**Table S2.** The gene list of novel targets prioritized by PandaOmics.

**Table S3.** The gene list of high confidence targets prioritized by PandaOmics.

**Table S4.** Expression profiles of GBP2, HCK, ICAM1, and ITGB2 in endometriosis bulk transcriptomics comparisons.

**Table S5.** Dysregulated pathways associated with GBP2, HCK, and ITGB2 in endometriosis comparisons.

**Table S6.** The gene list for target prioritization.

**Table S7.** Sequence of primers used for quantitative PCR.

**Supplementary video 1.** Target identification by PandaOmics.
